# MRI-based radiomics for noninvasive prediction of GPC3 expression in hepatocellular carcinoma: a systematic review and meta-analysis

**DOI:** 10.3389/fonc.2026.1771922

**Published:** 2026-09-11

**Authors:** Rui Gao, Xu Li

**Affiliations:** 1 Pingliang Hospital of Traditional Chinese Medicine (Integrated Traditional Chinese and Western Medicine Hospital), Pingliang, Gansu, China; 2 Gansu Medical College Affiliated Hospital, Pingliang, Gansu, China

**Keywords:** glypican-3 expression, hepatocellular carcinoma, MRI-based radiomics, noninvasive imaging biomarker, systematic review and meta-analysis

## Abstract

**Background:**

Hepatocellular carcinoma (HCC) is a leading cause of cancer death, and glypican-3 (GPC3) is a key diagnostic and therapeutic target currently assessed invasively. However, the overall diagnostic accuracy, between-study variability, and potential clinical relevance of Magnetic resonance imaging (MRI)-based radiomics models for predicting GPC3 expression have not been systematically quantified.

**Aim:**

Here, we systematically review and meta-analyze MRI-based radiomics models for noninvasive prediction of GPC3 expression in HCC.

**Methods:**

Studies developing or validating MRI-based radiomics models against histopathologic GPC3 expression were identified through database searches, and data on diagnostic accuracy, radiomics workflows, and study design were extracted for a bivariate random-effects meta-analysis and quality assessment using the adapted Quality Assessment of Diagnostic Accuracy Studies-2 (QUADAS-2) tool and the METhodological RadiomICs Score (METRICS) tool. Results: Of 63 records, seven retrospective studies comprising 1,209 patients met the inclusion criteria for the systematic review, of which five studies contributing eight model estimates were included in the quantitative synthesis. The pooled sensitivity was 0.81 (95% CI: 0.68–0.89), the pooled specificity was 0.87 (95% CI: 0.79–0.92), and the summary receiver operating characteristic curve yielded an area under the curve (AUC) of 0.91 (95% CI: 0.88–0.93). Sensitivity showed substantial heterogeneity (I² = 87.4%), whereas no heterogeneity was observed for specificity (I² = 0%). METRICS scores ranged from 56.1% to 92.2%.

**Conclusions:**

This systematic review and meta-analysis demonstrate that MRI-based radiomics provides a previously inaccessible, noninvasive window on GPC3 expression in HCC. The synthesis establishes a benchmark evidence base that opens the door to standardized radiomics pipelines, radiogenomic-guided trials, and imaging biomarkers to select patients for GPC3-targeted therapies.

## 1 Introduction

Hepatocellular carcinoma (HCC) is the most common primary liver malignancy and ranks among the leading causes of cancer-related death worldwide, reflecting its generally poor prognosis despite advances in surveillance and therapy (1, 2). The global burden of HCC closely mirrors the prevalence of chronic liver disease, particularly chronic hepatitis B and C, alcohol-related liver disease, and metabolic dysfunction–associated steatotic liver disease (2).

Glypican-3 (GPC3) is an oncofetal heparan sulfate proteoglycan that is frequently overexpressed in HCC, whereas its expression in normal adult liver is generally absent or low. However, focal or low-level expression has occasionally been reported in non-neoplastic settings, including cirrhotic or regenerative nodules, focal nodular hyperplasia, and active hepatitis (3, 4). Experimental and clinicopathologic studies indicate that GPC3 promotes tumor growth through pathways such as Wnt/β-catenin signaling, and its overexpression is associated with more aggressive tumor behavior (5, 6). Immunohistochemical analyses have further demonstrated that GPC3 expression correlates with poorer differentiation and reduced overall survival, supporting its role as an adverse prognostic biomarker in HCC (7).

Currently, GPC3 expression is primarily assessed by immunohistochemistry on biopsy or surgical resection specimens. Biopsy samples may not fully represent spatially heterogeneous tumors; in a prospective study of 100 liver nodules, the sensitivity of GPC3 immunohistochemistry decreased by 10.7% in needle-biopsy specimens relative to surgical specimens (8). Although percutaneous liver biopsy is generally safe, a multicenter study of 2,740 biopsies in patients with hepatitis C–related advanced fibrosis or cirrhosis reported serious adverse events in 1.1% and bleeding in 0.6% of procedures (9).These limitations support the investigation of noninvasive imaging surrogates that may characterize a larger tumor volume and can be repeated over time. Given that GPC3 is actively being explored as a target for antibody-based therapies, chimeric antigen receptor T-cell (CAR-T) products, and vaccine strategies, noninvasive methods to assess its expression could assist candidate selection for targeted treatment and the evaluation of biological heterogeneity over time. In this context, imaging-based surrogates of GPC3 status may help reduce the spatial sampling limitations of biopsy and better characterize intratumoral heterogeneity (10, 11).

Magnetic resonance imaging (MRI) is central to the noninvasive diagnosis and staging of HCC, and standardized systems such as Liver Imaging Reporting and Data System (LI-RADS) have improved lesion characterization in at-risk populations (12). Beyond conventional visual interpretation, MRI can interrogate multiple tissue properties through dynamic contrast enhancement, diffusion-weighted imaging, and hepatobiliary phase imaging, offering a rich substrate for quantitative image analysis (13). Radiomics, defined as the high-throughput extraction of quantitative features from medical images, enables computational characterization of tumor intensity, shape, and texture patterns that may reflect underlying histologic and molecular profiles (13).

In HCC, MRI-based radiomics has shown promise for tasks such as differential diagnosis, grading, prediction of microvascular invasion, and survival stratification, underscoring its potential as a precision oncology tool (13–15). Emerging radiogenomic studies suggest that radiomic signatures can capture gene expression programs and molecular phenotypes, raising the possibility of inferring biomarkers like GPC3 noninvasively (15, 16). Several recent investigations have developed radiomics models and nomograms using multisequence MRI to predict GPC3-positive HCC, reporting favorable discrimination and suggesting added value when radiomic features are combined with clinical and morphologic parameters (14–16).

Although individual studies have reported favorable performance, the available evidence remains limited by small retrospective cohorts and substantial heterogeneity in MRI acquisition, tumor segmentation, feature extraction, model development, and validation strategies. Consequently, the overall diagnostic accuracy of these models, the extent to which their performance is reproducible across different methodological settings, and their potential clinical relevance remain uncertain. A quantitative synthesis is therefore needed to estimate pooled diagnostic performance, characterize between-study heterogeneity, and evaluate how radiomics-based predictions may alter the estimated probability of GPC3 positivity. Accordingly, this systematic review and meta-analysis aimed to provide pooled estimates of the sensitivity, specificity, and area under the curve of MRI-based radiomics for the noninvasive prediction of GPC3 expression in HCC and to explore methodological and clinical factors that may contribute to heterogeneity in the reported performance.

## 2 Materials and methods

This review was conducted and reported in accordance with the PRISMA-DTA (Preferred Reporting Items for Systematic Reviews and Meta-Analyses of Diagnostic Test Accuracy Studies) guidelines (17).

### 2.1 Search strategy

Two reviewers independently performed a comprehensive literature search of PubMed, EMBASE, and Web of Science from database inception to December 2, 2025, using a predefined combination of terms related to radiomics and artificial intelligence (e.g., radiomics, radiomic, texture analysis, quantitative imaging, image features, feature extraction, deep learning, machine learning, artificial intelligence), MRI techniques (including conventional MRI, contrast-enhanced MRI, diffusion-weighted imaging, and apparent diffusion coefficient), GPC3 and its synonyms (such as glypican-3, GPC3 expression, GPC3 positivity, and related glypican terminology), and hepatocellular carcinoma (including MeSH terms and common variants such as hepatocellular carcinoma, HCC, liver cancer, primary liver tumor, and hepatic malignancy). All retrieved records were imported into Rayyan, where duplicates were removed before screening (18).

Two reviewers independently screened titles and abstracts to exclude irrelevant studies, followed by full-text assessment of potentially eligible articles; any disagreements were resolved through consultation and consensus between the two authors.

### 2.2 Inclusion and exclusion criteria

Study eligibility was determined using predefined inclusion and exclusion criteria based on a PICO framework, which were consistently applied to all potentially relevant full-text articles. The aim was to ensure that only methodologically sound and clinically relevant studies were incorporated into the review. Inclusion criteria were as follows: studies enrolling adult patients with histopathologically confirmed HCC; studies evaluating MRI-based radiomics models for the noninvasive prediction of GPC3 expression in HCC; and articles published in English. Studies in which HCC was diagnosed solely according to imaging criteria, including LI-RADS 5 lesions without histological confirmation, were not eligible. Additional exclusion criteria were as follows: non-English publications; studies that did not specifically investigate GPC3 expression in HCC; studies lacking a clearly described radiomics workflow (including, for example, image preprocessing, feature extraction, and model development) or not using quantitative imaging features derived through handcrafted or machine-based approaches; and non-original works such as case reports, review articles, conference abstracts, book chapters, commentaries, and letters.

### 2.3 Data extraction

Data were independently extracted by two reviewers using a standardized spreadsheet to ensure consistency across studies. Any discrepancies in data extraction were resolved through consultation and consensus between the two authors.

For each included study, the following information was collected, when reported: first author name, year of publication, and country of origin; study design (retrospective or prospective) and number of participating centers (single-center or multicenter); imaging characteristics, including MRI modality and magnetic field strength; reference standard used for confirming GPC3 expression; HCC characteristics, including the number of lesions and any reported lesion size cut-off; and patient demographics, including total sample size and the number of patients (or lesions, where applicable) allocated to the training, internal validation, and external validation cohorts.

Information on radiomics methodology was also systematically extracted using a structured template. For each study, data were collected across five domains of the radiomics workflow to allow for transparent comparison and synthesis. For segmentation, the following were recorded: segmentation method (manual, semi-automatic, or automatic), segmentation dimensionality (two-dimensional or three-dimensional), and the software used. When reported, we also extracted the procedures and metrics used to evaluate segmentation-related reproducibility, including intraobserver or interobserver assessment, intraclass correlation coefficient thresholds or values for radiomic features, and direct contour-agreement metrics such as the Dice similarity coefficient. For feature extraction, data included the types of features (e.g., first-order, texture, shape, or higher-order), the software platform used, and the total number of extracted features. For feature selection, the selection technique and the number of features retained after selection were documented. For the modeling domain, the overarching artificial intelligence paradigm (e.g., machine learning (ML) or deep learning (DL)) and the optimal algorithm reported in each study were extracted. Finally, information on nomogram construction was collected, including whether the radiomics model was combined with clinical variables and, if so, which clinical features were incorporated.

For quantitative synthesis, true-positive (TP), false-negative (FN), true-negative (TN), and false-positive (FP) counts were extracted directly when available. When raw contingency-table counts were not reported, they were reconstructed using the reported sensitivity, specificity, and numbers of GPC3-positive and GPC3-negative participants. TP and TN were calculated as TP=sensitivity×(N)positive and TN=specificity×N(negative)​, respectively, and rounded to the nearest whole number. FN and FP were subsequently calculated as FN=(N)positive​−TP and FP=(N)negative​−TN. The reconstructed sensitivity, specificity, and, when available, accuracy were back-calculated and compared with the published values, allowing for minor discrepancies attributable to rounding. AUC values alone were not used to reconstruct contingency tables because they do not represent diagnostic performance at a specific classification threshold. When cross-validation performance was reported without class-specific test-set denominators, the overall institutional cohort distribution was used as an approximation. The influence of this assumption was evaluated in a sensitivity analysis excluding the affected models.

### 2.4 Quality assessment

The methodological rigor of the included studies was evaluated using two complementary instruments: the METhodological RadiomICs Score (METRICS) Tool, version 1.0, and the Quality Assessment of Diagnostic Accuracy Studies-2 (QUADAS-2). To align QUADAS-2 with MRI-based radiomics studies, the signaling questions were adapted while retaining the original four domains and the standard risk-of-bias and applicability judgments (19). In the patient-selection domain, the adapted questions assessed alignment of the eligibility criteria with the review question, avoidance of two-gate case–control designs, consecutive or random enrolment, reporting of exclusions, and adequacy of clinical and demographic information. In the index-test domain, the questions addressed the reporting of MRI acquisition, tumor segmentation, image preprocessing, feature extraction, model validation, prespecification of classification thresholds, and blinding to the reference-standard results. The reference-standard domain evaluated histopathological and immunohistochemical confirmation of GPC3 expression, consistency of the antibody, scoring method and positivity threshold, and blinding to the radiomics results. The flow-and-timing domain assessed whether participants received the same reference standard, whether the interval between MRI and pathological assessment was appropriate, and whether exclusions, missing data, or incomplete validation could have introduced bias. The complete adapted signaling questions and response options are provided in **Supplementary Table 2**.

Concurrently, the METRICS Tool v1.0 was used to assess the quality, reproducibility, and transparency of the radiomics workflows. This tool comprises 30 items organized into nine methodological domains: study design, imaging data, image processing, feature extraction, metrics and comparison, testing procedures, feature processing, modeling preparation, segmentation, and open science. Items within each domain are hierarchically ordered according to methodological relevance. Item-level scores are expressed as percentages, and the overall methodological quality is graded as very low (0–19%), low (20–39%), moderate (40–59%), good (60–79%), or excellent (80–100%) (20). METRICS scores were generated using the publicly available web-based platform (https://metricsscore.github.io/metrics/METRICS.html).

### 2.5 Meta-analysis

The meta-analysis was conducted using Stata (version 15; StataCorp) and the MIDAS module to synthesize diagnostic test accuracy data. Pooled estimates of sensitivity, specificity, and their corresponding 95% confidence intervals (CIs) were calculated to quantify the overall diagnostic performance of MRI-based radiomics for predicting GPC3 expression. A summary receiver operating characteristic (SROC) curve was constructed, and the area under the curve (AUC) was derived to summarize the global discriminative ability of the included radiomics models. Forest plots of sensitivity and specificity were generated to depict between-study variation in diagnostic performance visually. Statistical heterogeneity was evaluated using the Cochran Q-test and the I^2^ statistic, with the following thresholds applied for interpretation: 0–25% (very low), 26–50% (low), 51–75% (moderate), and >75% (high heterogeneity).

To explore potential sources of heterogeneity and differences in diagnostic accuracy, subgroup factors were selected based on their plausible influence on MRI acquisition, radiomics workflow, clinical case mix, model construction, validation strategy, and methodological quality, as well as the availability of extractable data across the included studies. Subgroup analyses were performed according to eight study- or model-level covariates: MRI field strength (1.5 T vs. 3 T), use of 3D Slicer for tumor segmentation (3D Slicer vs. other software), use of PyRadiomics for feature extraction (PyRadiomics vs. other software), incorporation of clinical variables into the radiomics model (combined radiomics–clinical models vs. radiomics-only models), inclusion of alpha-fetoprotein (AFP) as a clinical variable (AFP-combined vs. non-AFP models), tumor burden (solitary HCC vs. non-solitary/multifocal disease), evaluation strategy (models tested on an independent validation set vs. models evaluated on the entire cohort without a separate validation set), and methodological quality based on METRICS (studies with an excellent METRICS rating vs. those with good or moderate scores).

A sensitivity analysis was performed by excluding studies for which diagnostic performance estimates were derived from the entire study cohort or were not based on an independently separable validation cohort, because these conditions could introduce optimism bias. The same bivariate random-effects model used in the primary analysis was applied to the remaining estimates. Deeks’ funnel plot asymmetry test was used to assess publication bias. In addition, the potential clinical utility of the radiomics models was examined using Fagan’s nomogram based on the pooled likelihood ratios. A pre-test probability of 25% was selected as an illustrative clinical scenario rather than as a validated or universally applicable prevalence estimate.

## 3 Results

### 3.1 Study selection and PRISMA flow diagram

**Figure 1:**
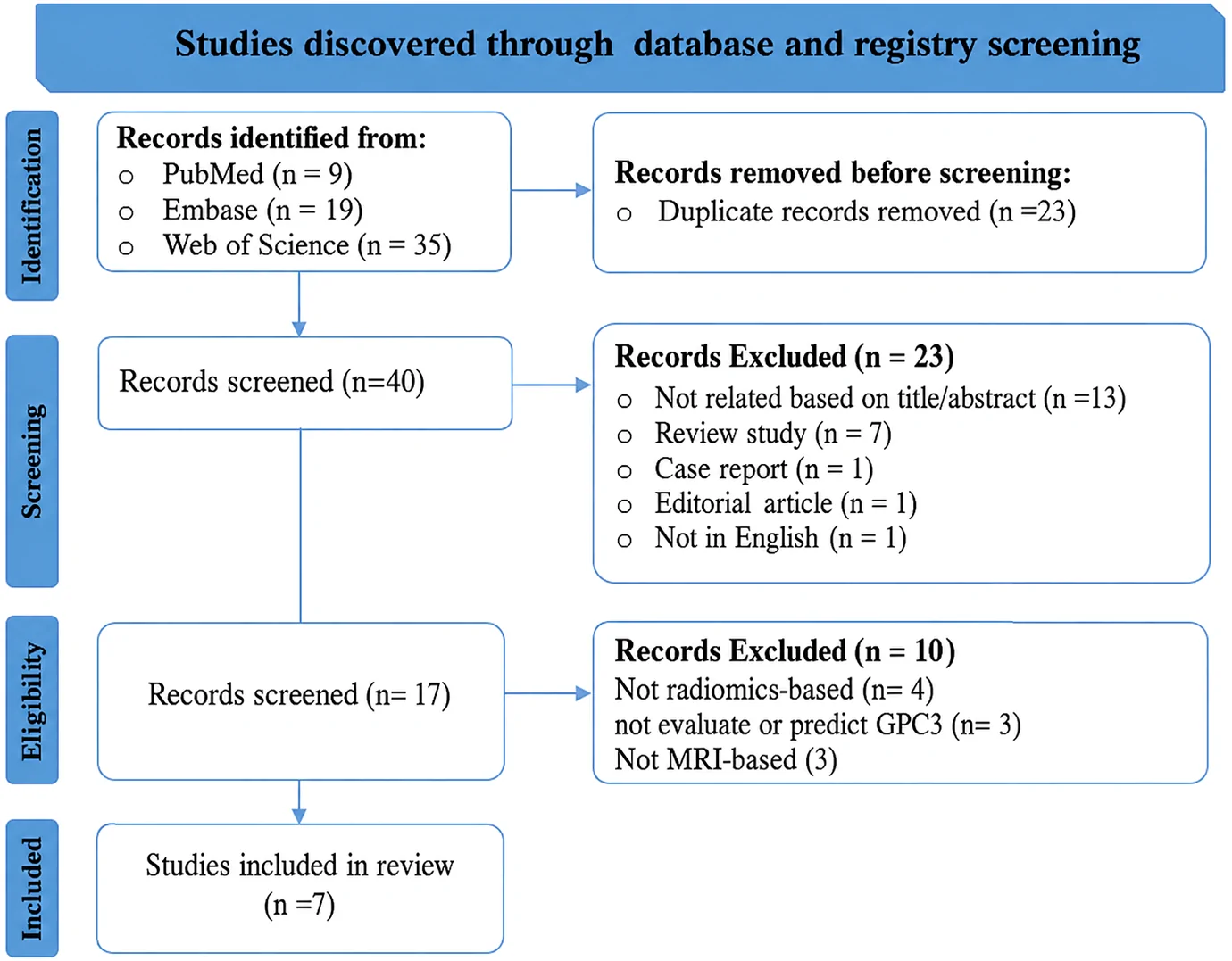
PRISMA flow diagram of study selection for MRI-based radiomics studies predicting glypican-3 (GPC3) expression in HCC.

Primary searches across the electronic databases yielded 63 records relevant to the research question. After removal of 23 duplicate entries, 40 unique records remained for title and abstract screening, as outlined in the PRISMA flow diagram in **Figure 1**. During this phase, 23 records were excluded because they were topically unrelated, were review articles, case reports, or editorial pieces, were not English studies, or otherwise failed to meet the basic eligibility criteria. The full texts of the remaining 17 articles were then evaluated in detail, leading to the exclusion of 10 studies that were not MRI-based, did not employ radiomics methods, or did not assess or predict GPC3 expression. Ultimately, seven studies met all predefined inclusion criteria and were included in the systematic review and qualitative synthesis, of which five studies contributing eight model estimates were included in the quantitative synthesis (21–27).

### 3.2 Quality assessment

**Figure 2:**
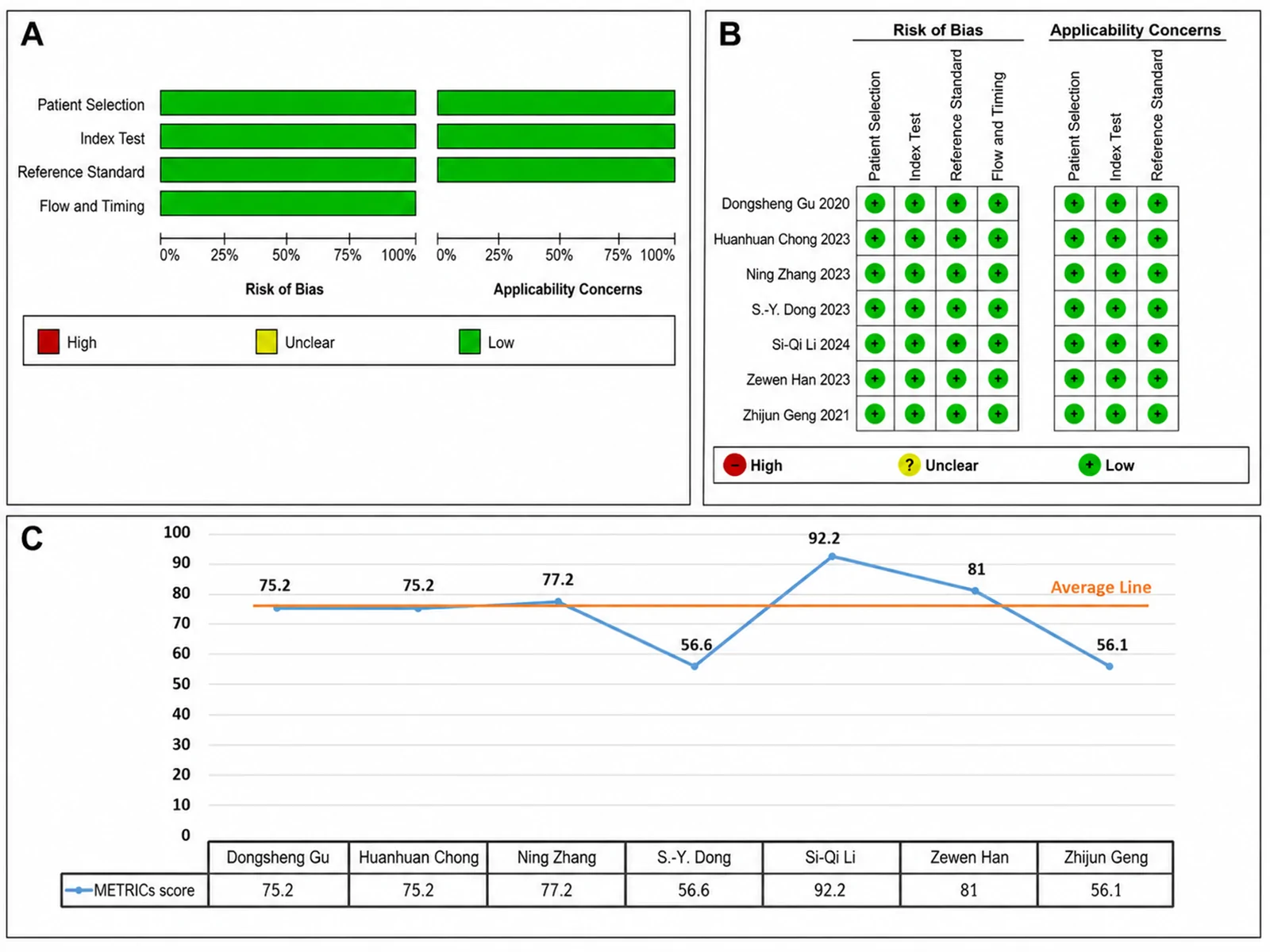
Quality assessment of the included studies. **(A)** Summary of risk-of-bias and applicability-concern judgments across the adapted QUADAS-2 domains. **(B)** Study-level QUADAS-2 traffic-light plot showing judgments for each included study. **(C)** METRICS methodological-quality scores for the included studies, with the horizontal line indicating the mean score.

Application of the adapted QUADAS-2 criteria resulted in a low risk-of-bias judgment for all seven studies across the patient-selection, index-test, reference-standard, and flow-and-timing domains. No applicability concerns were identified for patient selection, the MRI-based radiomics index test, or the pathological GPC3 reference standard. Study-level domain judgments are presented in **Figures 2A and B**, and the complete adapted signaling questions and decision criteria are provided in **Supplementary Table 2**.

Assessment with the METRICS tool showed methodological quality scores ranging from 56.1% to 92.2%. According to predefined METRICS thresholds, two studies were rated as having “excellent” quality (25, 26), three as “good” quality (21–23), and two as “moderate” quality (24, 27). The mean METRICS score across all included studies was 73.4%, consistent with an overall good methodological standard. A detailed, study-level breakdown of METRICS items and total scores is provided in the **Supplementary Material** (**Supplementary Table 1**), with an aggregated visual summary depicted in **Figure 2C**.

### 3.3 Data extraction

**Table 1** summarizes the key characteristics of the seven included studies, which were published between 2020 and 2024. All investigations were conducted in China and adopted a retrospective design. Six studies were single-center, while one study involved a multicenter cohort (25). MRI was the exclusive imaging modality across all studies. Of these, four studies acquired images on 1.5T scanners, whereas three studies used 3T systems (23, 26, 27). Histopathology with immunohistochemical staining for GPC3 expression served as the reference standard in all investigations.

**Table 1:** General information of included studies.

Study	Year	Country	Study design	Centers	Imaging		Gold standards	HCC type		Patients demographics (N)			
					Modality	Strength		Number	Size	Total	Training	Internal validation	External validation
Dongsheng Gu (21)	2020	China	R	Single	MRI	1.5 T	Histopathology with immunohistochemical	single-lesion	–	293	195	98	–
Huanhuan Chong (22)	2023	China	R	Single	MRI	1.5 T	Histopathology with immunohistochemical	–	HCC ≤ 5 cm	259	five-fold cross-validation		–
Ning Zhang (23)	2023	China	R	Single	MRI	3 T	Histopathology with immunohistochemical	single-lesion	No explicit upper size cutoff.	186	137	49	–
S.-Y. Dong (24)	2023	China	R	Single	MRI	1.5 T	Histopathology with immunohistochemical	–	HCC ≤ 3 cm	149	–	–	–
Si-Qi Li (25)	2024	China	R	Multi	MRI	1.5 T	Histopathology with immunohistochemical	–	–	143	five-fold cross-validation		20
Zewen Han (26)	2023	China	R	Single	MRI	3 T	Histopathology with immunohistochemical	single-lesion	No explicit upper size cutoff; CNLC stage Ia–IIa.	126	88	38	–
Zhijun Geng (27)	2021	China	R	Single	MRI	3 T	Histopathology with immunohistochemical	–	HCC > 1 cm	53	–	–	–

R, Retrospective; HCC, Hepatocellular carcinoma; N, Number.

Three studies restricted inclusion to patients with solitary HCC lesions (21, 23, 26), while the remaining studies also enrolled patients with multiple lesions. Size-related criteria were rechecked against the original reports. Only Chong et al. restricted inclusion to HCCs measuring ≤5 cm; Zhang et al. and Han et al. did not apply an explicit 5-cm upper-size cutoff, although Han et al. reported a subgroup analysis for tumors <5 cm. Mean or median tumor-size statistics were not consistently extractable across the included studies and were reported in heterogeneous formats; therefore, tumor size could not be formally evaluated as a moderator of model performance. In total, 1209 patients were included across the seven studies.

**Table 2** presents the radiomics workflow characteristics of the included studies. All seven studies performed manual three-dimensional tumor segmentation. Three investigations used 3D Slicer as the primary segmentation software, whereas others employed platforms such as ITK-SNAP (22), LIFEx (24), or the United Image Intelligent Research Platform System (25); one study did not report the segmentation software used (21).

**Table 2:** Radiomics characteristics data.

Study	Segmentation			Features extraction			Features selection		Model		Nomogram	
	Method	Dimension	Software	Types	Software	Number	Selection method	(N) after selection	AI base	Optimal algorithm	Combined	Clinical features
Dongsheng Gu	Manual	3D	NR	Shape, first-order, texture, and wavelet-based features.​	NR	853	ICC > 0.75 Mann–Whitney U test Fisher score	10	ML	SVM	Yes	AFP
Huanhuan Chong	Manual	3D	ITK-SNAP	Shape, first-order, and texture features.​	PyRadiomics	749	ICC > 0.8 t test LASSO, RF	10	ML	LR	Yes	AFP, Homogeneous T2 signal, HBP hypointensity
Ning Zhang	Manual	3D	3D Slicer	Shape, first-order, texture, and wavelet-based features.​	PyRadiomics	1,688	ICC > 0.8 t test LASSO	11	ML	LR	Yes	Age, AFP, Non-smooth tumor margin
S.-Y. Dong	Manual	3D	LIFEx	Shape, first-order, texture, and wavelet-based features.​	LIFEx	NR	Univariate logistic regression Multivariate logistic regression	3	ML	LR	Yes	AFP
Si-Qi Li	Manual	3D	United Image Intelligent	Shape, first-order, texture, and wavelet-based features.​	United Image Intelligent	16,337	ICC > 0.8 Pearson correlation, LASSO	10	ML	LR	Yes	tumor morphology microvascular invasion
Zewen Han	Manual	3D	3D Slicer	Shape, first-order, and texture features.​	PyRadiomics	2,782	ICC > 0.8 mRMR 10-fold cross-validation	8	ML	LR	Yes	AFP
Zhijun Geng	Manual	3D	3D Slicer	Shape, first-order, and texture features.​	PyRadiomics	107	ICC ≥0.75 retained Spearman correlation	1	ML	LR	No	–

NR, Not reported; N, Number; 3D, 3-dimensional; AFP, Alpha-fetoprotein; ICC, Intraclass correlation coefficient; LASSO, Least Absolute Shrinkage and Selection Operator; RF, Random forest; mRMR, Minimum Redundancy Maximum Relevance; ML, Machine learning; LR, Logistic regression; SVM, Support vector machine.

Across studies, radiomics features commonly encompassed shape, first-order, texture, and wavelet-based descriptors. Four studies extracted features using PyRadiomics, while others used LIFEx or the United Image Intelligent Research Platform System; one study did not specify the feature-extraction software (21).

Feature selection strategies varied among studies and are detailed in **Table 2**. Six studies performed ICC-based assessments of radiomic-feature reproducibility after repeated manual segmentation, whereas one study did not report an ICC assessment (24). The reported feature-retention thresholds ranged from 0.75 to 0.80. Only one study additionally reported direct contour agreement using the Dice similarity coefficient, with a mean value greater than 0.90 across five MRI phases; the mean ICC of radiomic features in that study was greater than 0.80.

All included studies adopted ML–based approaches for model development. Logistic regression (LR) was the predominant optimal classifier, reported in six studies, while one study used a support vector machine (SVM) as the final model (21). Six studies constructed combined nomogram models integrating radiomics signatures with clinical variables, whereas one study relied on a radiomics-only model (27). AFP was the most frequently incorporated clinical parameter, included in five studies; additional clinical variables used for model construction are summarized in **Table 2**.

### 3.4 Meta-analysis

For each included study, a 2×2 contingency table (TP, FP, TN, FN) was constructed from the validation cohorts using the reported or derivable sensitivity, specificity, and sample size data. Of the seven studies, two did not provide sufficient information to reconstruct a complete 2×2 table and were therefore excluded from the quantitative meta-analysis (22, 24). In two additional studies that did not report a separate validation cohort, the entire study population was used to estimate TP, FP, TN, and FN (25, 27). Five of the seven included publications contributed data to the quantitative synthesis. Because Gu et al., Li et al., and Han et al. (21, 25, 26). each reported two eligible models, whereas Zhang et al. and Geng et al. (23, 27). each reported one eligible model, these five publications contributed eight model estimates in total. Accordingly, the unit counted in the pooled and subgroup analyses was the model estimate rather than the publication. Consistent with best practice, only diagnostic performance metrics derived from validation cohorts were used to populate the 2×2 tables, when available, to minimize the risk of overfitting and inflation of diagnostic performance estimates. Details of the reconstructed 2×2 contingency tables are summarized in **Table 3**.

**Table 3:** Reported and reconstructed 2×22\times22×2 contingency tables used in the quantitative synthesis.

Study	Group	Model	AUC	Sens	Spec	Total analyzed(n)	GPC3 + (n)	GPC3 - (n)	TP	FN	TN	FP
Dongsheng Gu	validation cohort	Radiomics	0.871	0.721	0.833	98	68	30	49	19	25	5
Dongsheng Gu	validation cohort	Combined	0.914	0.735	0.933	98	68	30	50	18	28	2
Ning Zhang	validation cohort	Combined	0.8	0.585	1	49	41	8	24	17	8	0
Si-Qi Li †	total cohort	Radiomics	0.979	0.947	0.8	123	98	25	93	5	20	5
Si-Qi Li †	total cohort	Combined	0.989	0.944	0.8	123	98	25	93	5	20	5
Zewen Han	validation cohort	Radiomics	0.851	0.828	0.889	38	29	9	24	5	8	1
Zewen Han	validation cohort	Combined	0.862	0.793	0.778	38	29	9	23	6	7	2
Zhijun Geng	total cohort	Radiomics	0.76	0.575	0.923	53	40	13	23	17	12	1

†For Li et al., sensitivity and specificity were reported for cross-validation test results, whereas the exact class-specific test-set denominators were not provided. The 2×22\times22×2 counts were therefore approximated using the reported GPC3-positive and GPC3-negative distribution of the institutional cohort and nearest-integer rounding.

AUC, Area Under the Curve; Sens, Sensitivity; Spec, Specificity; n, Number; TP, True positive; FN, False negative; TN, True negative; FP, False positive.

#### 3.4.1 Model accuracy

**Figure 3:**
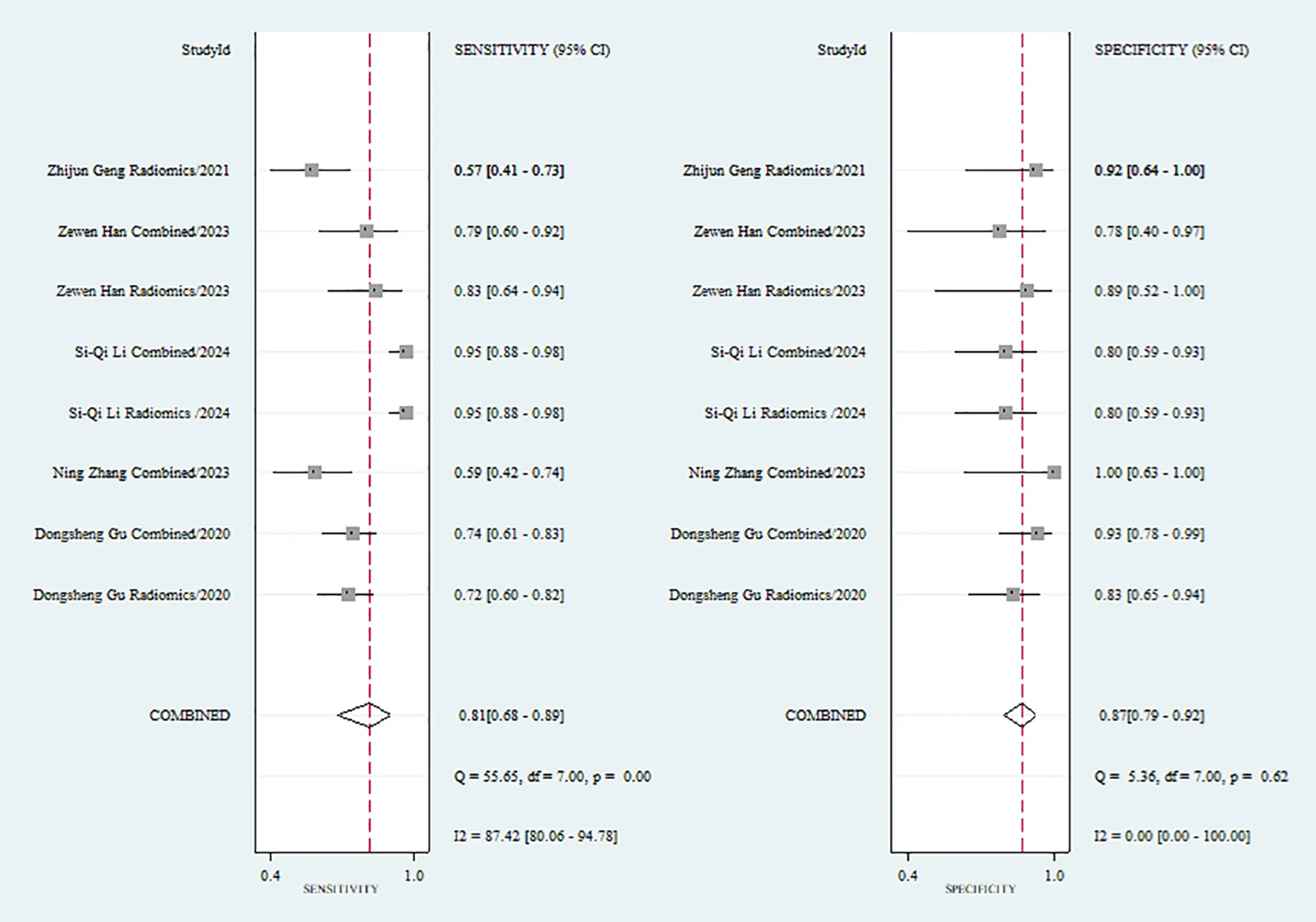
Forest plots showing study-level and pooled sensitivity and specificity.

The meta-analysis included eight distinct radiomics-based models evaluating the diagnostic performance of MRI-derived radiomics for predicting GPC3 expression in HCC. The pooled sensitivity was 0.81 (95% CI: 0.68–0.89), and the pooled specificity was 0.87 (95% CI: 0.79–0.92), as illustrated in **Figure 3**.

**Figure 4:**
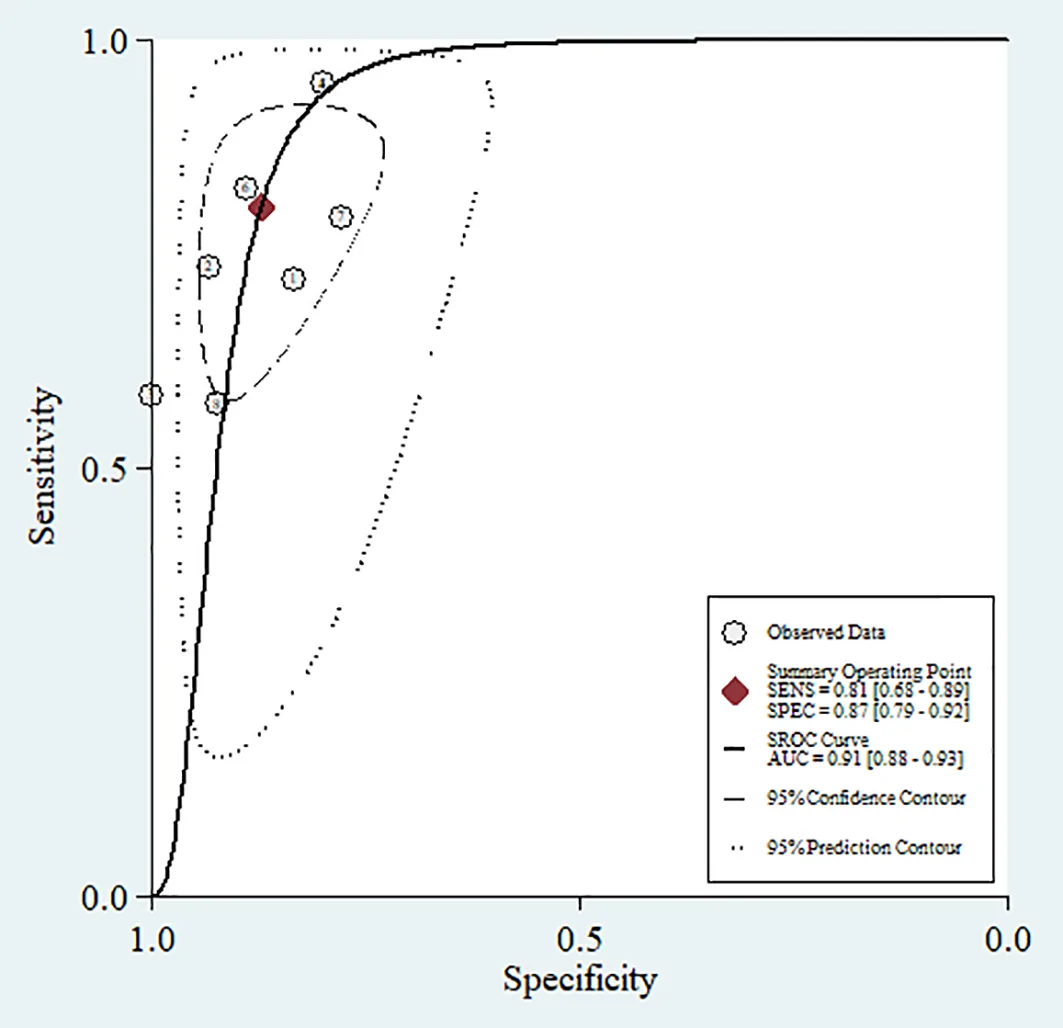
SROC curve for MRI-based radiomics models predicting GPC3 expression in HCC.

Assessment of heterogeneity demonstrated substantial between-study variability in sensitivity (I² = 87.4%, 95% CI: 80.1–94.8%, p < 0.001), whereas no heterogeneity was observed for specificity (I² = 0.0%, 95% CI: 0.0–100.0%, p = 0.62). The SROC curve yielded an AUC of 0.91 (95% CI: 0.88–0.93), indicating excellent overall discriminative ability of radiomics models for identifying GPC3-positive HCC. The corresponding ROC curve is presented in **Figure 4**.

#### 3.4.2 Sensitivity analysis

After excluding the estimates from Li et al. and Geng et al. (25, 27), five model estimates from three studies remained in the sensitivity analysis. The pooled sensitivity decreased to 0.73 (95% CI: 0.65–0.79), while the pooled specificity was 0.89 (95% CI: 0.79–0.94). The summary ROC analysis yielded an AUC of 0.87 (95% CI: 0.84–0.89). Heterogeneity was moderate for sensitivity (I^2^ = 35.82%, Q = 6.23, p = 0.18) and absent for specificity (I^2^ = 0.00%, Q = 3.50, p = 0.48). Compared with the primary analysis, exclusion of these studies reduced the pooled sensitivity and SROC AUC but produced a similar pooled specificity. Sensitivity heterogeneity also decreased substantially, suggesting that studies without independently evaluable validation data contributed to the variability and possible optimism of the primary estimates.

#### 3.4.3 Publication bias and clinical utility analysis

**Figure 5:**
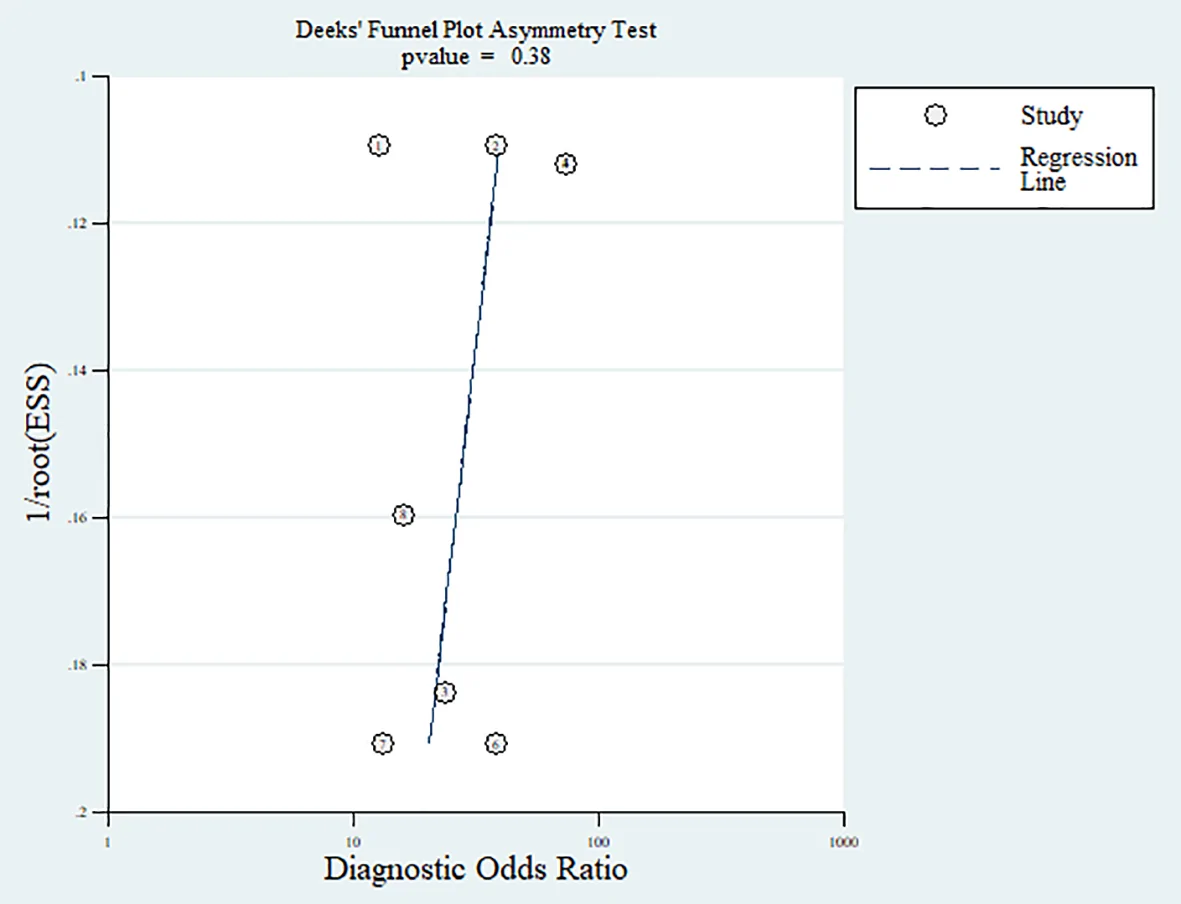
Deeks’ funnel plot for assessment of publication bias.

**Figure 6:**
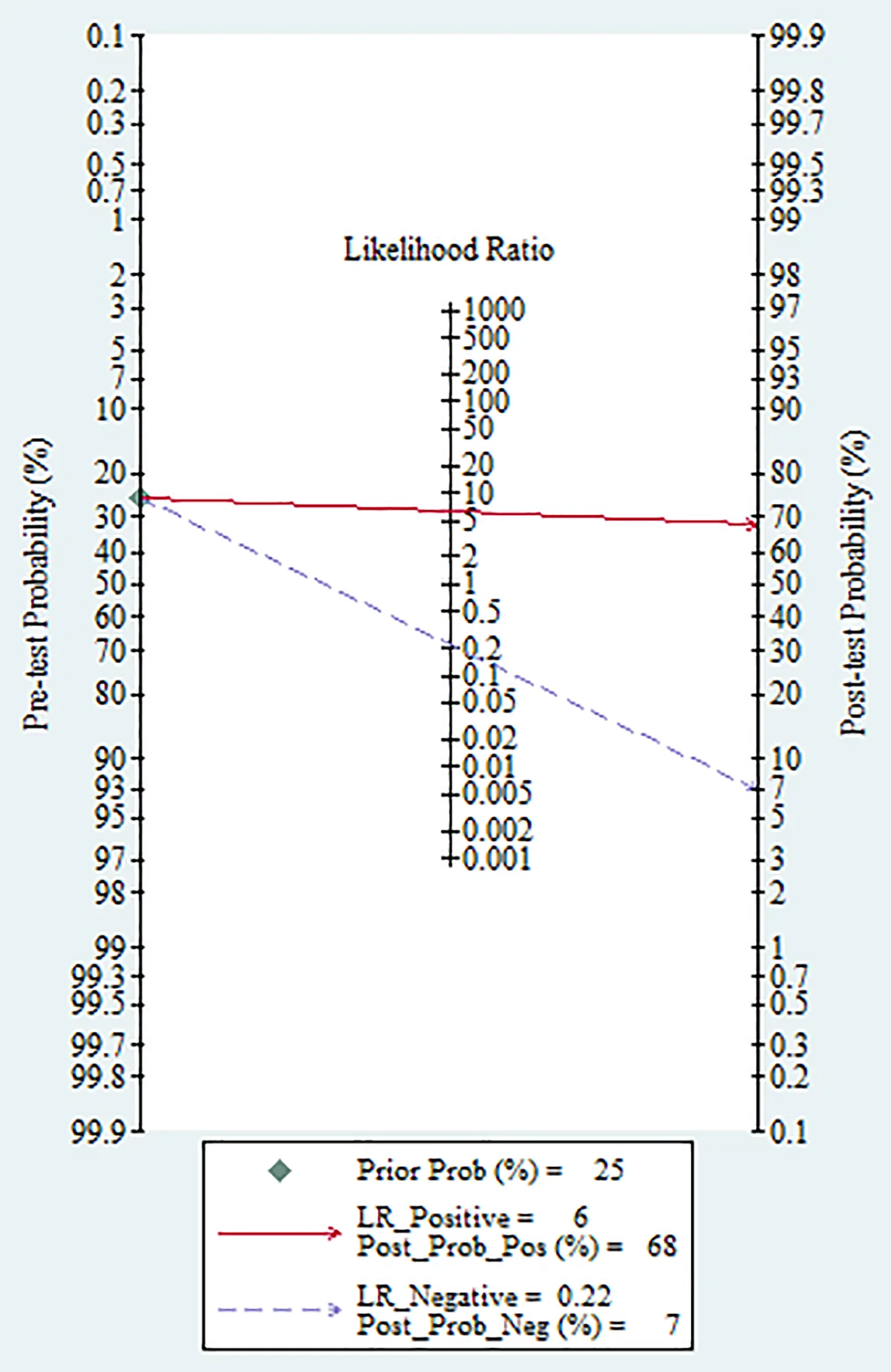
Fagan nomogram demonstrating how MRI-based radiomics assessment of GPC3 expression modifies post-test probability across different pre-test probabilities.

**Figure 5** presents Deeks’ funnel plot for assessing publication bias, which showed no significant small-study effect among the included radiomics studies (p = 0.38). Clinical utility was further evaluated using a Fagan nomogram (**Figure 6**). Assuming a pre-test probability of 25%, a positive radiomics prediction increased the post-test probability of GPC3-positive HCC to 68%, corresponding to a positive likelihood ratio (PLR) of 6.0. Conversely, a negative radiomics result reduced the post-test probability to 7% and yielded a negative likelihood ratio (NLR) of 0.22. These findings suggest that MRI-based radiomics models provide meaningful incremental value for noninvasive risk stratification of GPC3 expression in HCC.

#### 3.4.4 Subgroup analysis

Although heterogeneity was high for sensitivity and absent for specificity, subgroup analyses were performed to explore potential sources of variability in diagnostic performance across study- and model-level characteristics. These subgroup analyses were planned *a priori* to evaluate whether specific methodological or clinical factors modified test accuracy estimates. They were conducted using the following prespecified covariates: 1) MRI field strength (1.5 T vs. 3 T), 2) use of 3D Slicer for tumor segmentation (3D Slicer vs. other software), 3) use of PyRadiomics for feature extraction (PyRadiomics vs. other software), 4) incorporation of clinical variables into the radiomics model (combined radiomics–clinical models vs. radiomics-only models), 5) inclusion of AFP as a clinical variable (AFP-combined vs. non-AFP models), 6) tumor burden (solitary vs. non-solitary/multifocal HCC), 7) model evaluation strategy (independent validation set vs. no separate validation set), and 8) methodological quality based on METRICS (excellent vs. good/moderate). The results of these subgroup analyses are presented in **Table 4**.

**Table 4:** Subgroup analysis of diagnostic performance across different covariates.

Covariate		Number of model estimates	Sensitivity	Specificity
MRI Field Strength	1.5 T	4	0.87	0.90
	3 T	4	0.71	0.85
Tumor Burden	Solitary HCC	5	0.74	0.88
	Non-solitary HCC	3	0.88	0.85
Incorporation of clinical variables	Combined	4	0.81	0.89
	Radiomics	4	0.80	0.86
AFP as a clinical variable	Incorporated AFP	5	0.74	0.88
	Did not include AFP	3	0.88	0.85
Segmentation software	3D Slicer	4	0.71	0.90
	Others	4	0.87	0.85
Methodological quality	Excellent	4	0.91	0.82
	Moderate or good	4	0.67	0.90
Evaluation strategy	Independent validation	5	0.74	0.88
	Entire cohort	3	0.88	0.85
Feature extraction software	PyRadiomics	4	0.71	0.90
	Other	4	0.87	0.85

MRI, Magnetic resonance imaging; 3D, 3-dimensional; HCC, Hepatocellular carcinoma; AFP, Alpha-fetoprotein.

Given the small number of model estimates within each subgroup, the overlap between several confidence intervals, and the inclusion of correlated estimates derived from the same study cohorts, all between-group comparisons were considered exploratory and should not be interpreted as definitive evidence of independent covariate effects.

##### 3.4.4.1 MRI field strength

For the MRI field strength subgroup, models developed on 1.5 T scanners (4 studies) achieved a pooled sensitivity of 0.87 (95% CI: 0.78–0.96) and a specificity of 0.85 (95% CI: 0.77–0.92). In contrast, models based on 3 T MRI (4 studies) showed lower sensitivity (0.71; 95% CI: 0.54–0.87) but higher specificity (0.90; 95% CI: 0.80–1.00).​ The between-group comparison indicated a statistically significant difference in sensitivity, favoring 1.5 T over 3 T (p = 0.02), whereas the difference in specificity between 1.5 T and 3 T was not statistically significant (p = 0.59). Although the between-group test was statistically significant for sensitivity, this exploratory finding does not establish that 1.5 T MRI is superior to 3 T MRI.

##### 3.4.4.2 Tumor burden

For the lesion number subgroup, models developed in cohorts restricted to solitary HCC (5 studies) achieved a pooled sensitivity of 0.74 (95% CI: 0.60–0.86) and specificity of 0.88 (95% CI: 0.81–0.96). In contrast, studies including non-solitary or multifocal disease (3 studies) demonstrated a higher sensitivity of 0.88 (95% CI: 0.78–0.98) but a slightly lower specificity of 0.85 (95% CI: 0.74–0.96).​ The between-group comparison showed a statistically significant difference in sensitivity, favoring models developed in non-solitary cohorts (p = 0.03). In contrast, the difference in specificity between solitary and non-solitary groups was not statistically significant (p = 0.34). The apparent difference in sensitivity may reflect differences in disease severity, tumor burden, or cohort composition rather than an independent effect of lesion number.

##### 3.4.4.3 Incorporation of clinical variables

In the subgroup analysis comparing combined radiomics–clinical models with radiomics-only models, studies that integrated clinical variables (4 studies) achieved a pooled sensitivity of 0.81 (95% CI: 0.66–0.95) and specificity of 0.89 (95% CI: 0.81–0.97). Radiomics-only models (4 studies) showed a very similar sensitivity of 0.80 (95% CI: 0.66–0.95) and a slightly lower specificity of 0.86 (95% CI: 0.76–0.95).​ Between-group comparisons indicated no statistically significant differences in sensitivity (p = 0.51) or specificity (p = 0.34) between combined and radiomics-only models. The available data did not demonstrate a clear improvement in sensitivity or specificity after incorporating routine clinical variables into radiomics models.

##### 3.4.4.4 Inclusion of AFP as a clinical variable

In the subgroup analysis evaluating the impact of AFP, models that incorporated AFP alongside radiomics features (5 studies) achieved a pooled sensitivity of 0.74 (95% CI: 0.60–0.88) and specificity of 0.88 (95% CI: 0.81–0.97). In contrast, models that did not include AFP as a predictor (3 studies) demonstrated a higher sensitivity of 0.88 (95% CI: 0.78–0.98) but a slightly lower specificity of 0.85 (95% CI: 0.76–0.95).​ The between-group comparison showed a statistically significant difference in sensitivity, favoring non-AFP models (p = 0.03), whereas the difference in specificity between AFP-combined and non-AFP models was not statistically significant (p = 0.34). Although sensitivity was lower among models incorporating AFP, this exploratory subgroup finding should not be interpreted as evidence that AFP has a detrimental effect on model performance. The observed difference may reflect variation in cohort characteristics, model construction, or the handling of correlated predictors rather than an independent effect of AFP inclusion.

##### 3.4.4.5 Methodological quality (METRICS)

In the subgroup analysis by methodological quality, studies rated as excellent on the METRICS tool (4 studies) had a pooled sensitivity of 0.91 (95% CI: 0.87–0.96) and a specificity of 0.82 (95% CI: 0.72–0.91). Studies with good or moderate METRICS ratings (4 studies) showed a lower pooled sensitivity of 0.67 (95% CI: 0.57–0.76) but a higher specificity of 0.90 (95% CI: 0.84–0.97). Between-group comparisons indicated no statistically significant difference in sensitivity between excellent and good/moderate quality studies (p = 0.68), whereas specificity was significantly higher in the good/moderate group (p = 0.00). These findings suggest that higher methodological quality is associated with greater sensitivity of MRI-based radiomics models for predicting GPC3 expression. In contrast, studies with slightly lower quality scores tend to report more conservative but more specific models.

### 3.4.4.6 Tumor segmentation software

In the subgroup defined by segmentation software, studies that used 3D Slicer for tumor delineation (4 studies) reported a pooled sensitivity of 0.71 (95% CI: 0.54–0.87) and a specificity of 0.90 (95% CI: 0.80–1.00). Studies that employed other segmentation platforms (4 studies) demonstrated a higher sensitivity of 0.87 (95% CI: 0.78–0.96) but a slightly lower specificity of 0.85 (95% CI: 0.77–0.92). Between-group comparisons showed a statistically significant difference in sensitivity, favoring non–3D Slicer software (p = 0.02), whereas the difference in specificity between 3D Slicer and other tools was not statistically significant (p = 0.59). These differences should not be interpreted as a direct effect of methodological-quality category, because METRICS ratings may also reflect differences in validation strategy, software, and model complexity.

### 3.4.4.7 Model evaluation strategy (independent validation vs. entire cohort)

In the subgroup analysis by evaluation strategy, models that were tested on an independent validation set (5 studies) achieved a pooled sensitivity of 0.74 (95% CI: 0.60–0.86) and a specificity of 0.88 (95% CI: 0.81–0.96). Models evaluated on the entire cohort without a separate validation set (3 studies) demonstrated a higher sensitivity of 0.88 (95% CI: 0.78–0.98) but a slightly lower specificity of 0.85 (95% CI: 0.74–0.96).​ The between-group comparison showed a statistically significant difference in sensitivity, favoring models without independent validation (p = 0.03), while the difference in specificity was not statistically significant (p = 0.34). The observed difference cannot be attributed specifically to segmentation software, because software choice was likely associated with other differences in radiomics workflow.

### 3.4.4.8 Feature extraction software

In the subgroup analysis based on feature extraction software, studies that used PyRadiomics (4 studies) achieved a pooled sensitivity of 0.71 (95% CI: 0.54–0.87) and a specificity of 0.90 (95% CI: 0.80–1.00). In contrast, studies relying on alternative feature extraction tools (4 studies) demonstrated a higher sensitivity of 0.87 (95% CI: 0.78–0.96) but a slightly lower specificity of 0.85 (95% CI: 0.77–0.92).​ The between-group comparison showed a statistically significant difference in sensitivity, favoring non-PyRadiomics software (p = 0.02), whereas the difference in specificity between PyRadiomics and other tools was not statistically significant (p = 0.59). The observed difference should not be interpreted as evidence that one feature-extraction platform is superior, because software choice may be confounded by differences in preprocessing and model-development procedures.

## 4 Discussion

Hepatocellular carcinoma is a major cause of cancer-related death worldwide and often presents at advanced stages despite improved surveillance in at-risk populations. Glypican-3 is an oncofetal cell-surface proteoglycan that is largely absent in normal adult liver but overexpressed in most HCCs, where its expression is associated with more aggressive disease and poorer prognosis, making it an attractive diagnostic and therapeutic target. In this context, MRI-based radiomics, which extracts quantitative features from routine liver MRI beyond visual assessment, offers a promising noninvasive approach to capture GPC3-related tumor phenotypes and support image-based molecular profiling in HCC (15, 28, 29).

In this meta-analysis, MRI-based radiomics models for predicting GPC3 expression in HCC achieved a pooled sensitivity of 0.81 and specificity of 0.87, with an AUC of 0.91, indicating excellent overall discriminative performance for identifying GPC3-positive tumors. The corresponding positive and negative likelihood ratios of 6.0 and 0.20 translated, in Fagan nomogram analysis, into an increase in post-test probability from 25% to 68% after a positive test and a reduction to 6% after a negative test, underscoring the capacity of these models to shift estimated probability of GPC3 positivity in both directions meaningfully. Collectively, these findings suggest that MRI-derived radiomics can provide robust noninvasive estimation of GPC3 status and may serve as a valuable tool for image-based molecular phenotyping and candidate selection for GPC3-targeted strategies in HCC.

The sensitivity analysis excluding Li et al. and Geng et al. produced lower pooled sensitivity and SROC AUC than the primary analysis, while pooled specificity remained comparable. The substantial reduction in sensitivity heterogeneity indicates that estimates derived without independently evaluable validation data may have contributed to both optimism and between-model variability. Nevertheless, the sensitivity-analysis AUC of 0.87 indicated that the overall discriminatory performance remained reasonably strong. Univariate meta-regression was considered but was not performed because only five independent studies contributed to the quantitative synthesis, and several studies reported correlated model estimates from the same patient cohorts, making covariate-effect estimates underpowered and potentially unstable.

The Fagan analysis illustrates how MRI-based radiomics may modify the estimated probability of GPC3 expression; however, its clinical interpretation depends on the assumed pre-test probability. The 25% pre-test probability used in the MIDAS analysis was selected as an illustrative scenario rather than derived as a validated prevalence estimate for patients being considered for GPC3-targeted therapy. At this assumed pre-test probability, a positive result increased the post-test probability of GPC3 expression to 68%, whereas a negative result reduced it to 7%. Although these probability shifts may assist in risk stratification and prioritization for confirmatory assessment, a post-test probability of 68% is not sufficient as a standalone basis for GPC3-targeted treatment selection because substantial uncertainty regarding true GPC3 expression remains. MRI-based radiomics should therefore be considered a complementary risk-stratification tool rather than a replacement for histopathological or immunohistochemical confirmation when verified GPC3 expression is required.

Previous HCC radiomics meta-analyses provide useful contextual benchmarks for the present findings; however, their performance estimates are not directly comparable because they evaluated different clinical endpoints, imaging modalities, model categories, and statistical performance measures. Jin et al. (30) showed that radiomics and combined radiomics–clinical models achieve C-indices around 0.79–0.83 for recurrence prediction on MRI, clearly outperforming clinical models alone, while Qin et al. (31) reported a pooled sensitivity of 0.72, specificity 0.88, and AUC 0.89 for MRI-based prediction of CK19-positive HCC using traditional, radiomics, and combined feature sets. Similarly, Gou et al. (32) demonstrated that radiomics models yield higher diagnostic accuracy than non-radiomics models for preoperative assessment of microvascular invasion in solitary HCC, again highlighting the added value of quantitative imaging over conventional approaches. Despite these methodological and clinical differences, the previous meta-analyses provide broader contextual support for the potential of imaging-based models to predict clinically relevant HCC characteristics. The numerical similarities should not be interpreted as evidence that GPC3-focused radiomics models are equivalent or superior to models developed for recurrence, CK19 expression, or microvascular invasion.

Methodological quality of the included radiomics studies was generally high, as reflected by uniformly low risk of bias and absence of applicability concerns across all QUADAS-2 domains, indicating appropriate patient selection, index test conduct, reference standards, and flow/timing for the review question. Complementary assessment with the METRICS tool yielded scores ranging from 56.1 to 92.2, with two studies rated excellent, three good, and two moderate, and a mean score of 73.4, consistent with an overall good standard of radiomics reporting and workflow transparency. These findings suggest that, despite some variability, the body of evidence underpinning MRI-based radiomics for GPC3 prediction rests largely on studies with acceptable to high methodological rigor, supporting confidence in the pooled diagnostic estimates.

Subgroup analyses were conducted to explore potential sources of the substantial heterogeneity in sensitivity estimates. Nominal between-group differences were observed across several technical and study-level characteristics, including MRI field strength, segmentation and feature-extraction software, tumor burden, AFP inclusion, methodological quality, and validation strategy. However, these comparisons were based on small numbers of model estimates, several confidence intervals overlapped, and some estimates originated from the same study cohorts. Moreover, many of the examined characteristics were interrelated; for example, scanner field strength, segmentation software, feature-extraction platform, and validation design may have varied together across studies. Consequently, the observed subgroup differences cannot be attributed confidently to any single technical or clinical factor and should be regarded as exploratory and hypothesis-generating.

The higher sensitivity observed among models evaluated without a separate validation cohort was consistent with possible optimism bias and aligned with the findings of the sensitivity analysis. In particular, the apparently higher sensitivity in the 1.5 T subgroup should not be interpreted as evidence that 1.5 T MRI is inherently superior to 3 T MRI. No direct within-study comparison of the two field strengths was available, and the observed difference may reflect residual confounding by sample size, scanner manufacturer, sequence, contrast-agent protocols, cohort characteristics, methodological quality, software selection, or validation strategy. Similar caution applies to the other subgroup patterns, which may reflect differences in cohort composition or the broader radiomics workflow rather than independent covariate effects. Larger, independent studies using standardized acquisition, segmentation, feature extraction, and validation procedures are required to determine whether these subgroup patterns represent reproducible effects.

The apparent difference associated with AFP inclusion should also not be interpreted as evidence that AFP adversely affects prediction. Although AFP is an established HCC biomarker, its incremental value for predicting GPC3 expression may depend on its relationship with the selected radiomic features and other clinical variables. Correlation or collinearity between predictors, differences in variable-selection procedures, small development samples, and inconsistent multivariable model adjustment could reduce the apparent added value of AFP or produce unstable performance estimates. Because the included studies did not provide sufficient standardized information to evaluate these mechanisms directly, this explanation remains hypothetical.

Taken together, these findings indicate that MRI-based radiomics provides a robust, noninvasive means of estimating GPC3 expression in HCC, with diagnostic performance comparable to that of established radiomics applications for other biological and prognostic endpoints. By demonstrating high specificity across heterogeneous technical settings and clinically meaningful post-test probability shifts, this meta-analysis supports the potential incorporation of GPC3-focused radiomics models into contemporary HCC care pathways to aid molecular phenotyping and targeted therapy selection, while also highlighting concrete avenues for methodological refinement and standardization in future studies.

### 4.1 Limitations

This systematic review and meta-analysis have several important limitations that should be considered when interpreting the findings. Foremost, the evidence base is still relatively small: despite a comprehensive search of three major databases from inception to December 2, 2025, and application of broad radiomics- and MRI-related terms, only seven studies met the strict eligibility criteria, and only five contributed sufficient data for quantitative pooling. This constrained number of studies and eight analyzable models primarily reflects the early, highly specialized nature of MRI-based radiomics for GPC3 in HCC, rather than an inadequate search or overly restrictive selection, because most excluded articles either did not use MRI, did not implement a complete radiomics workflow, or did not report GPC3-related outcomes in a manner suitable for diagnostic accuracy synthesis. As a result, the pooled estimates should be viewed as a first quantitative benchmark for this emerging field and will require confirmation as more high-quality studies become available.

The characteristics of the primary studies also limit generalizability. All seven investigations were retrospective and conducted in China, frequently in single-center settings. At least one included cohort consisted entirely of patients with HBV-related HCC, whereas the distribution of underlying liver disease etiologies was not consistently reported across the remaining studies. Consequently, the pooled estimates may predominantly reflect HBV-associated HCC and may not be directly generalizable to populations in which HCV, metabolic dysfunction-associated steatotic liver disease/NASH, or alcohol-related liver disease are more prevalent. An etiology-specific subgroup analysis was not feasible because the required data were incompletely and inconsistently reported. Validation in geographically and etiologically diverse populations is therefore required.

Although the total pooled sample of 1209 patients is nontrivial, individual studies often enrolled modest cohorts, sometimes with additional restrictions such as solitary lesions or specific tumor size thresholds, which can introduce selection bias and limit the generalizability of model performance to patients with multifocal or more advanced disease. Moreover, all studies relied on immunohistochemical assessment of GPC3 from surgical or biopsy specimens as the reference standard, which remains subject to sampling error and may not fully represent intratumoral heterogeneity, particularly in multifocal HCC.

Mean or median tumor size was not consistently reported across the included studies, precluding formal evaluation of tumor size as a moderator of radiomic feature stability or diagnostic performance.

Technical characteristics of the radiomics workflows represent another limitation. All included studies relied on manual three-dimensional tumor segmentation, which is labor-intensive and potentially sensitive to interobserver variation. Although six studies used ICC-based criteria to identify radiomic features that remained reproducible after repeated segmentation, these analyses primarily assessed feature stability rather than direct spatial agreement between tumor contours. Only one study reported a direct contour-overlap metric, with a mean Dice similarity coefficient greater than 0.90 across five MRI phases. The absence of consistently reported contour-agreement measures and complete ICC distributions prevented quantitative synthesis and limits assessment of segmentation reproducibility across readers, institutions, and imaging settings. In addition, the developed models predominantly used conventional machine-learning classifiers, most commonly LR, with limited exploration of more advanced algorithms, which may restrict the ability to fully capture complex imaging–biological relationships and potentially underestimate the performance ceiling of MRI-based radiomics for GPC3 prediction.

Overall, these limitations indicate that, while the current synthesis provides an important early evidence map and demonstrates encouraging performance of MRI-based radiomics for noninvasive GPC3 prediction, the results should be interpreted as provisional and hypothesis-generating. They highlight an urgent need for larger, multicenter, prospectively designed, and transparently reported studies across diverse populations and imaging environments.

For two models, class-specific denominators corresponding to the reported cross-validation test-set performance were unavailable; therefore, the 2×2 counts were approximated using the overall institutional cohort distribution, which may have introduced minor imprecision into the pooled estimates.

### 4.2 Future directions

Future research should expand and diversify the evidence base for MRI-based radiomics in GPC3-positive HCC through larger, prospective, multicenter studies that enroll patients across diverse regions, etiologies, and disease stages, including multifocal and advanced tumors. Such trials should incorporate standardized MRI acquisition protocols, clearly defined and reproducible radiomics pipelines, and routine use of external validation cohorts to generate performance estimates that are both robust and clinically generalizable. Given that current models rely heavily on manual three-dimensional segmentation and predominantly use logistic regression, future studies should evaluate semi-automatic and automatic segmentation methods and benchmark architectures specifically suited to multisequence MRI. These may include convolutional neural networks for spatial feature learning, attention-based or transformer architectures for integrating complementary information across MRI sequences and contrast-enhancement phases, and transfer-learning or foundation-model approaches pretrained through supervised or self-supervised learning on large MRI or medical-imaging datasets. Such approaches should be compared directly with conventional handcrafted radiomics and radiomics–clinical models using independent external validation, calibration analysis, and decision-curve analysis, with transparent reporting under METRICS and PRISMA-DTA–aligned guidelines.

In parallel, integrating radiomics with other biomarker domains and clinical endpoints will be critical for translation. Studies that combine GPC3-focused radiomic signatures with genomic, transcriptomic, or liquid biopsy data and assess longitudinal imaging changes in the context of systemic, locoregional, or GPC3-targeted therapies could clarify whether these models can serve not only as baseline stratification tools but also as dynamic markers for treatment selection and response.

## 5 Conclusion

This systematic review and meta-analysis suggests that MRI-based radiomics shows promising ability to noninvasively estimate GPC3 expression in hepatocellular carcinoma, with a pooled sensitivity of 0.81, specificity of 0.87, and AUC of 0.91. However, these estimates should be interpreted cautiously because they were derived from a preliminary evidence base comprising seven retrospective studies conducted in a single country, with substantial heterogeneity in sensitivity. Across diverse technical settings, specificity remained consistently high. Exploratory subgroup analyses suggested possible differences in sensitivity according to MRI field strength, segmentation and feature-extraction software, tumor burden, and validation strategy. However, these findings were based on small subgroup samples and require confirmation in larger, independent datasets. Although the included evidence derives mainly from retrospective, single-country studies, overall methodological quality was generally good, supporting cautious confidence in these pooled estimates while underscoring that they should be viewed as preliminary benchmarks. Collectively, these findings support further evaluation of MRI-based radiomic signatures as potential tools for image-based molecular phenotyping and GPC3-related risk stratification. Larger, prospective, geographically diverse, multicenter studies with rigorous external validation are required before these models can be considered sufficiently reliable for clinical decision-making.

## Data Availability

The original contributions presented in the study are included in the article/**Supplementary Material**. Further inquiries can be directed to the corresponding author.
