# Supplementary material for: MRI-based radiomics for noninvasive prediction of GPC3 expression in hepatocellular carcinoma: a systematic review and meta-analysis
Source: Front Oncol. 2026 Sep 11;16:1771922. doi: 10.3389/fonc.2026.1771922 (PMC13612196; doi:10.3389/fonc.2026.1771922)
Supplement: Supplementary file 1 [file SupplementaryFile1.docx]

**Supplementary S2. Adapted QUADAS-2 signaling questions and decision criteria for MRI-based radiomics studies predicting GPC3 expression.**

**Patient Selection**

1. **Were the inclusion and exclusion criteria explicitly defined and aligned with the review scope (e.g., patients with pathologically confirmed hepatocellular carcinoma who underwent preoperative MRI, excluding recurrent or metastasized HCC cases)? (Yes/No/Unclear)**
2. **Was the study design clearly described (prospective cohort, retrospective cohort, nested case–control, etc.) and free of obvious two-gate (case–control) bias? (Yes/No/Unclear)**
3. **Were patients enrolled consecutively or randomly (rather than by convenience sampling), and were any a priori exclusions (e.g., poor MRI image quality, incomplete clinical data) reported? (Yes/No/Unclear)**
4. **Were key demographics and clinical characteristics adequately reported (age, sex, tumor size, AFP level, liver function, underlying etiology)? (Yes/No/Unclear)**
5. **Are there concerns that the included patients and setting do not match the review question (e.g., inclusion of different tumor types or inappropriate imaging modalities)? (High concern/Low concern/Unclear concern)**

**Index Test (Radiomics Model)**

6. **Was the MRI acquisition and radiomics feature extraction process clearly described (MRI sequence, imaging parameters, segmentation method, feature extraction software, preprocessing steps)? (Yes/No/Unclear)**
7. **Was a validation method used (pre-specified cut-off, external validation cohort, or internal cross-validation rather than post-hoc model adjustment)? (Yes/No/Unclear)**
8. **Did the application of the index test align with this review’s objective (prediction of GPC3 expression in HCC using MRI-based radiomics)? (Yes/No/Unclear)**
9. **Were index-test interpreters (radiomics modellers) blinded to the reference-standard results (e.g., GPC3 immunohistochemistry), and was the positivity threshold pre-specified? (Yes/No/Unclear)**
10. **Are there concerns that the index test, its conduct, or interpretation differs from the review question (e.g., inadequate feature selection, differing target biomarker)? (High concern/Low concern/Unclear concern)**

**Reference Standard (GPC3 Expression Assessment)**

11. **Was the definition of the target condition (GPC3-positive hepatocellular carcinoma confirmed by immunohistochemistry or molecular analysis) consistent with the review objectives? (Yes/No/Unclear)**
12. **Were reference-standard interpreters blinded to the index-test (radiomics) results? (Yes/No/Unclear)**
13. **Was the same reference standard (e.g., GPC3 IHC scoring method, antibody used, positivity threshold) applied to all participants? (Yes/No/Unclear)**
14. **Are there concerns that the target condition, as defined by the reference standard, does not match the review question? (High concern/Low concern/Unclear concern)**

**Flow and Timing**

15. **Were all individuals assessed using an identical reference standard for GPC3 evaluation? (Yes/No/Unclear)**
16. **Was the time interval between MRI and pathological GPC3 assessment appropriate to minimize bias? (Yes/No/Unclear)**
17. **Could the patient flow (exclusions, missing data, or incomplete model validation) have introduced bias? (High risk/Low risk/Unclear risk)**
